# Olfactory functional covariance connectivity in Parkinson’s disease: Evidence from a Chinese population

**DOI:** 10.3389/fnagi.2022.1071520

**Published:** 2023-01-04

**Authors:** Shouyun Du, Yiqing Wang, Guodong Li, Hongyu Wei, Hongjie Yan, Xiaojing Li, Yijie Wu, Jianbing Zhu, Yi Wang, Zenglin Cai, Nizhuan Wang

**Affiliations:** ^1^Department of Neurology, Guanyun County People's Hospital, Lianyungang, China; ^2^Department of Neurology, The Affiliated Suzhou Hospital of Nanjing University Medical School, Suzhou, China; ^3^Department of Neurology, Suzhou Science & Technology Town Hospital, Gusu School, Nanjing Medical University, Suzhou, China; ^4^Department of Neurology, Affiliated Lianyungang Hospital of Xuzhou Medical University, Lianyungang, China; ^5^Department of Radiology, The Affiliated Suzhou Hospital of Nanjing University Medical School, Suzhou, China; ^6^School of Biomedical Engineering, ShanghaiTech University, Shanghai, China

**Keywords:** Parkinson’s disease, functional covariance connectivity, resting-state fMRI, gray matter, white matter, olfactory function

## Abstract

**Introduction:**

Central anosmia is a potential marker of the prodrome and progression of Parkinson’s disease (PD). Resting-state functional magnetic resonance imaging studies have shown that olfactory dysfunction is related to abnormal changes in central olfactory-related structures in patients with early PD.

**Methods:**

This study, which was conducted at Guanyun People’s Hospital, analyzed the resting-state functional magnetic resonance data using the functional covariance connection strength method to decode the functional connectivity between the white–gray matter in a Chinese population comprising 14 patients with PD and 13 controls.

**Results:**

The following correlations were observed in patients with PD: specific gray matter areas related to smell (i.e., the brainstem, right cerebellum, right temporal fusiform cortex, bilateral superior temporal gyrus, right Insula, left frontal pole and right superior parietal lobule) had abnormal connections with white matter fiber bundles (i.e., the left posterior thalamic radiation, bilateral posterior corona radiata, bilateral superior corona radiata and right superior longitudinal fasciculus); the connection between the brainstem [region of interest (ROI) 1] and right cerebellum (ROI2) showed a strong correlation. Right posterior corona radiation (ROI11) showed a strong correlation with part 2 of the Unified Parkinson’s Disease Rating Scale, and right superior longitudinal fasciculus (ROI14) showed a strong correlation with parts 1, 2, and 3 of the Unified Parkinson’s Disease Rating Scale and Hoehn and Yahr Scale.

**Discussion:**

The characteristics of olfactory-related brain networks can be potentially used as neuroimaging biomarkers for characterizing PD states. In the future, dynamic testing of olfactory function may help improve the accuracy and specificity of olfactory dysfunction in the diagnosis of neurodegenerative diseases.

## 1. Introduction

Recently, resting-state functional magnetic resonance imaging (fMRI) has found widespread application for the analysis of various stages of Parkinson’s disease (PD). fMRI analysis for PD encompasses regional homogeneity (ReHo; Zang et al., 2004), amplitude of low-frequency fluctuation (ALFF; Han et al., 2011), and functional connectivity (Olde Dubbelink et al., 2014). Independent component analysis of resting-state fMRI can identify several specific brain networks in the awake and resting states, such as the default mode, salience, and executive control networks, which have been widely investigated in a variety of neurodegenerative diseases, including PD (Wang and Weiming, 2012; Wang et al., 2013, 2015, 2016a,b; Pellegrino et al., 2016; Chang et al., 2017; Shi et al., 2017; Xiong et al., 2021).

Central anosmia may be a potential marker of the prodrome and disease progression in PD and Alzheimer’s disease (AD; Turetsky et al., 2009; Zou et al., 2015). In 2017, Fjaeldstad et al (Fjaeldstad et al., 2017) developed a new method of structural olfactory connectivity fingerprinting to study two functional and structural maps of the olfactory cortical network and constructed a combined map containing the expected structural connectivity, which can be used as a potential neuroimaging biomarker of early structural connectivity changes in diseases such as PD. In 2021, we developed a new spectral contrast mapping method to decode brain activity at the voxel level, and analyzed 15 patients with severe hyposmia, 15 patients with no/mild hyposmia, and 15 healthy controls. Patients with severe or no/mild hyposmia presented with prominent differences in the vermis, cerebellum, and insula, while patients with severe hyposmia showed differences in the frontal, parietal, and temporal lobe gyri compared to the healthy control group (Yu et al., 2021).

Although some progress has been made in brain structural and fMRI studies for PD, the changes in the connectivity of the white matter and gray matter related to the olfactory system have received little attention. Mounting evidence shows that the blood-oxygen-level-dependent imaging (BOLD) signal in the white matter undergoes stimulus-related synchronous changes in response to olfactory and other related stimuli, and the signal changes in white matter pathway possess specificity (Ding et al., 2018). White-gray matter functional covariance connectivity can explore differences between brain functions. For example, Chen et al. used a white-gray matter functional connectivity approach to explore brain function in autistic and normal children (Chen et al., 2021). The ALFF of resting-state fMRI signals reflects the intensity of regional spontaneous brain activity (Zou et al., 2008), and there is a specific frequency distribution of amplitude low-frequency BOLD fluctuations in gray matter and white matter (Peer et al., 2017). We adopted a new ALFF-based functional covariance connectivity method for gray and white matter to explore PD-associated imaging markers. Our previous study showed that (Wang et al., 2022) the functional covariance connection strength (FCS) values of the dorsolateral prefrontal cortex of the right hemisphere and superior corona radiata of the left hemisphere are independent risk factors for PD, which may be helpful to explore the early pathogenesis of PD. Thus, we hypothesized that the olfactory-related gray–white matter connectivity information of patients with PD would be specific. This study aimed to analyzed the resting-state functional magnetic resonance data using the FCS method to decode the functional connectivity between the white–gray matter in a Chinese population.

## 2. Materials and methods

### 2.1. Participants

This study enrolled 14 patients with primary PD admitted to Guanyun County People’s Hospital between June 2020 and June 2022. All patients with PD were diagnosed by two chief neurologists. The inclusion criteria were as follows: (1) patients diagnosed with PD according to the Movement Disorder Society’s clinical diagnostic criteria for PD (Postuma et al., 2015), (2) the diagnosis was consistent with the findings of clinical examination and complete scale and imaging evaluations, and (3) patients who provided written informed consent. The exclusion criteria were as follows: (1) patients whose Parkinson’s symptoms were caused by definite infection, trauma, overdose of drugs, poisoning and other factors in the past; (2) patients with a past history of intracranial nucleus destruction surgery, deep brain electrical stimulation implantation, transcranial magnetic stimulation (TMS), and other treatments; (3) patients with severe heart failure, respiratory failure, renal insufficiency, and other diseases; (4) patients diagnosed with psychiatric diseases (such as anxiety disorder, depression, etc.); (5) patients with stroke (lesion diameter > 20 mm), intracranial space occupying lesions, and other diseases; and (6) patients with other conditions deemed unsuitable for research. At the same time, 15 age-and sex-matched normal controls [healthy control (HC) group] were recruited. The normal controls provided written informed consent and did not meet the exclusion criteria. This study was approved by the Ethics Committee of Suzhou Science and Technology Town Hospital.

### 2.2. Clinical assessment measures

Demographic and clinical information such as sex, age at admission, age at onset, first symptom (tremor or rigidity), disease duration, and other data of all patients with PD were collected by one neurologist. All the different domains and the entirety of the Movement Disorder Society Unified Parkinson’s Disease Rating Scale [including part I (non-motor experiences of daily living), part II (motor aspects of daily living), part III (motor examination), and part IV (motor complications)] and the Hoehn and Yahr Scale were used to evaluate disease severity. All patients were evaluated by trained clinicians.

### 2.3. MRI acquisition

Patients underwent imaging with a 1.5-T MRI System (OPTIMA MR360; GE, Milwaukee, WI). T1-sequences were acquired using the following scanning parameters: three-dimensional magnetization-prepared rapid gradient echo sequences, repetition time (TR) = 1904.26 ms, echo time (TE) = 28.20 ms, inversion time (TI) =750.00 ms, thickness = 5 mm, flip angle (FA)  = 90°, matrix size = 288 × 224, and 32 sagittal slices. Resting-state fMRI was performed with the following parameters: TR =3,000 s, TE = 40 ms, TI = 0.00 ms, 32 transverse slices, layer spacing = 5 mm, thickness = 3 mm, matrix size = 64 × 64, and FA = 90°. The total resting-state fMRI scanning time was 384 s. fMRI scanning was conducted in darkness, and participants were given clear instructions to relax, close their eyes and not fall asleep during MR acquisition in the resting state (confirmed immediately after the experiment). Earplugs were used to reduce scanner noise and cushions were used to minimize head movement.

### 2.4. Preprocessing of fMRI data

The resting-state fMRI data were preprocessed according to our previous research (Wang et al., 2022) using DPABI (http://rfmri.org/dpabi; Yan et al., 2016), which is based on Statistical Parametric Mapping (SPM12; https://www.fil.ion.ucl.ac.uk/spm/) and the Resting-State fMRI Data Analysis Toolkit (REST, Song et al., 2011; http://www.restfmri.net). The default method selected for this study is called “New Segment + DARTEL” in SPM8. The Friston 24 parameter model was selected to adjust for the effect of head movement. We used Peer et al.’s method (Peer et al., 2017) to filter the band of gray matter, whose fMRI image signal was 0.01–0.1 Hz, while that of the white matter was 0.01–0.15 Hz. The resultant images were spatially normalized to the Montreal Neurological Institute echo-planar imaging template using default settings and resampling to 3 × 3 × 3 mm^3^ voxels, and smoothed with a Gaussian kernel of 4 × 4 × 4 mm^3^.

**Figure 1:**
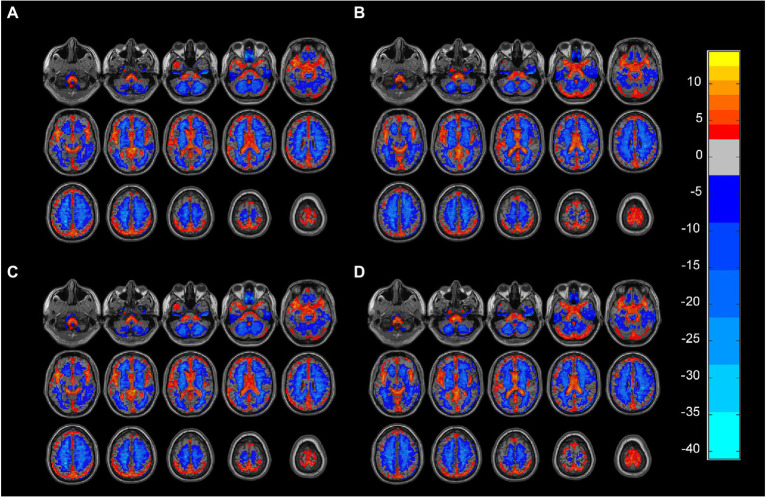
**(A)** Gray matter ALFF mapping of PD group (One-sample *t*-test, FDR, *p* < 0.05). **(B)** Gray matter ALFF mapping of HC group (One-sample *t*-test, FDR, *p* < 0.05). **(C)** White matter ALFF mapping in the PD group (One-sample *t*-test, FDR, *p* < 0.05). **(D)** White matter ALFF mapping in the PD group (One-sample *t*-test, FDR, *p* < 0.05). ALFF: amplitude of low-frequency fluctuations, PD: Parkinsons’s disease, HC: healthy control.

We adopted the white matter and gray matter masks in MRIcroN as the group mask.^1^ Individual mapping distinguishes gray matter from white matter in the group mask to avoid voxels that overlap between the two. In this study, the matrix dot product operation in MATLAB was used to remove the mixed signals from the images.^2^ The signals of the non-white matter and non-gray matter were set to zero in the ALFF atlas. ALFF maps of gray matter and white matter were obtained for each participant (Wang et al., 2022). We performed a one-way ANOVA on the four groups of ALFF profiles obtained as shown in Figure 1. All image operations were performed over an area measuring 61 × 73 × 61 mm^3^.

The FCS value was calculated for each participant (Wang et al., 2022). Drawing on the definition of structural covariance linkage (Eisenberg et al., 2015), the functional covariance connection was defined as the Pearson correlation coefficient between the ALFF values of two voxels for all participants, and was calculated using the following formula:


(1) $$r=\frac{1}{N-1}\overset{N}{\underset{i=1}{\sum }}\left(\frac{X_{i}-\bar{X}}{S_{X}}\right)\left(\frac{Y_{i}-\bar{Y}}{S_{Y}}\right)=\frac{1}{N-1}\overset{N}{\underset{i=1}{\sum }}Z_{\mathrm{Xi}}Z_{\mathrm{Yi}},$$

where N is the number of participants, $\bar{X}$ and $\bar{Y}$ are the mean values of the data X and Y, and S is the sample standard deviation. The Pearson correlation coefficient (r) between the data series X and Y can be represented as the normalized inner product of standard scores (z-scores). We use the product ( $Z_{\mathrm{Xi}}Z_{\mathrm{Yi}}$ ) of the z-score of each individual (i) as a measure of the strength of the individual’s functional connection. The FCS is the product of the standard scores of each pair of voxels. The FCS can be used to measure the strength of the functional covariance of each pair of voxels (Eisenberg et al., 2015; Chen et al., 2021).

### 2.5. Obtaining regions of interest

We obtained a voxel-based 67,541 × 1,632 functional covariance connection matrix (67,541 represents the number of gray matter voxels, and 1,632 represents the number of white matter voxels) for each participant. This study tested whether each gray–white matter connection differed significantly between the PD and HC groups by comparing the functional covariance contribution values using t-tests, whose significance was set at *p* < 0.00005. Areas with differences in gray matter–white matter connection relative to the controls were considered as regions of interest (ROIs).

### 2.6. Correlation analysis of ROI and Parkinson’s scale

The functional connections between the gray and white matter of the whole brain were constructed. The functional gray–white matter connection with *p* < 0.00005 was denoted as a brain area with a significant difference. The DPABI V6.1 ROI signal extractor tool was used to extract the signal value of this area with the coordinates as the origin and a radius of 3 mm to analyze the correlation between the nine gray matter brain areas.16 spherical ROIs were drawn. We analyzed the correlation of the nine brain regions, to obtain the correlation coefficient and map them. Thereafter, the respective correlations between the signal values of the nine ROIs and the subscores of each domain of the Unified Parkinson’s Scale were plotted and analyzed.

## 3. Results

### 3.1. Demographic comparison between the two groups

Initially, 18 patients with PD were included in this study, of which 4 were excluded from the statistical analysis due to considerable head movement and poor image data quality, while 5 participants from the control group were not included in the statistical analysis for the same reasons. Finally, 14 patients with PD (8 men, 6 women; mean age, 67.2 ± 10.1 years; range, 46–80 years) who had not received anti-Parkinsonian medication or were taking only levodopa (L-DOPA) and 13 HCs (4 men, 9 women; age, 69.9 ± 6.4 years; range, 61–79 years) were enrolled in the study. There were no significant differences in sex and age between the two groups (*p* < 0.05; Table 1). All participants were right-handed.

**Table 1:** Baseline data.

Clinical characteristics	Patients with Parkinson’s disease	Healthy controls	*p* Value
All patients	14	13	
Sex			0.252
Women	6 (40%)	9 (60%)	
Men	8 (66.7%)	4 (33.3%)	
Age ± SD	67.2 ± 10.1	69.9 ± 6.4	0.081

### 3.2. Abnormal functional covariance connections for the HC and PD groups

**Figure 2:**
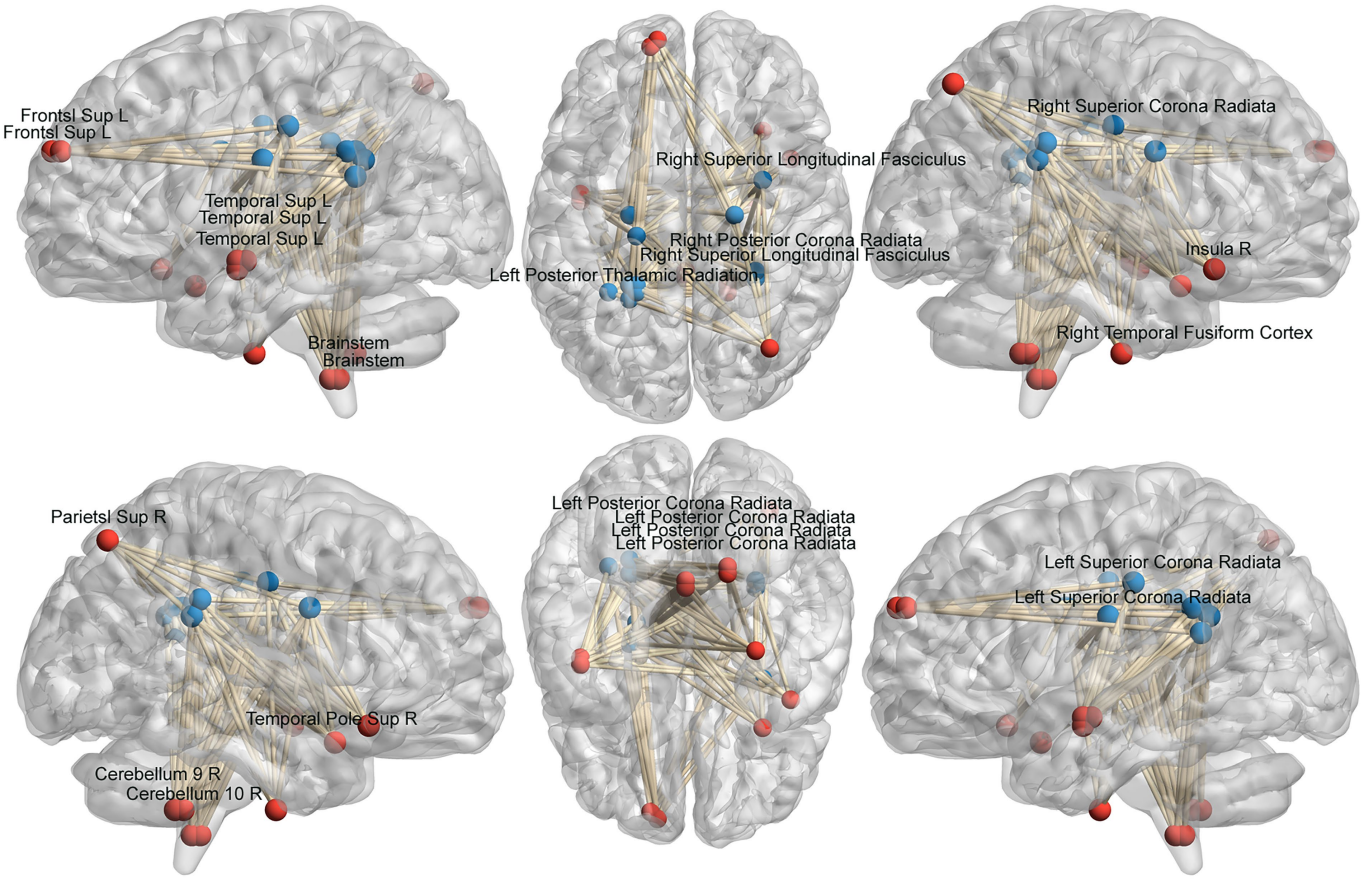
Schematic diagram of the abnormal functional covariance connections between 13 gray matter voxels and 11 white matter voxels identified by FCS analysis for the PD and HC groups. The red balls represent gray matter voxels, and the blue balls are the white matter voxels. PD: Parkinsons’s disease, HC: healthy control.

We analyzed the voxel-based 67,541 × 1,632 functional covariance connection matrix, where 67,541 represents the number of gray matter voxels and 1,632 represents the number of white matter voxels. Subsequently, FCS analysis revealed that 13 × 11 functional covariance connections differed significantly between the PD and control groups (Figure 2).

### 3.3. Analysis of cerebral gray matter ROIs

**Figure 3:**
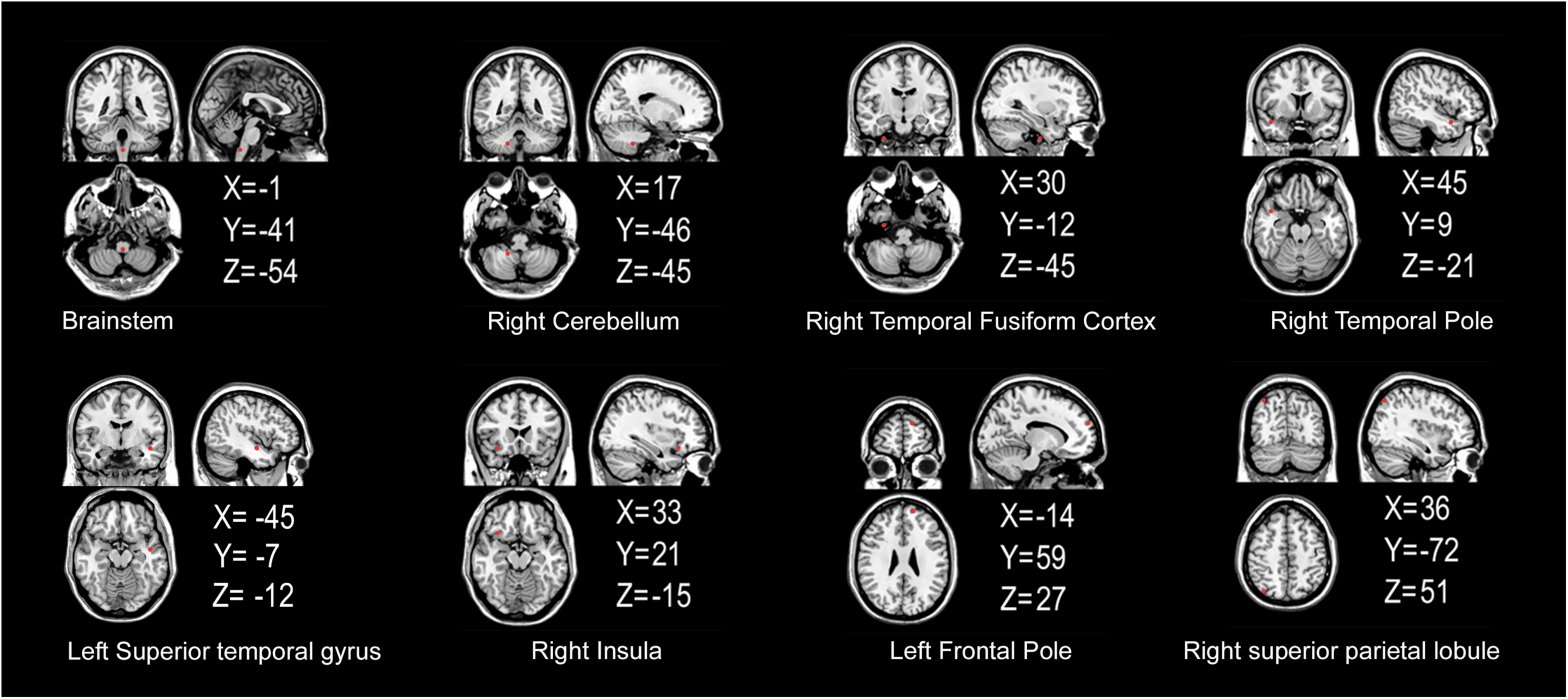
Gray matter areas with abnormal functional covariance connections. The areas marked in red represent the gray matter areas, where the FCS is statistically different between the PD and HC groups (two sample *T*-test, *p* < 0.00005). X, Y, and Z refer to the coordinate axes in the Montreal Neurological Institute space. PD: Parkinsons’s disease, HC: healthy control, FCS: functional covariance connection strength.

The FCS of 13 gray matter areas in the PD group differed from that of the control group (Table 2). They were mainly located in the brainstem, right cerebellum, right temporal fusiform cortex, bilateral superior temporal gyrus, right Insula, left frontal pole, and right superior parietal lobule (Table 3; Figure 3).

**Table 2:** Gray matter brain areas with significant differences between the PD and control groups based on anatomical automatic labeling.

Coordinates			Anatomical automatic labeling
X	Y	Z	
0	−42	−54	Brainstem
0	−39	−54	Brainstem
18	−48	−45	Cerebellum_9_R
18	−45	−45	Cerebellum_10_R
30	−12	−45	Right Temporal Fusiform Cortex
45	9	−21	Temporal_Pole_Sup_R
−45	−6	−15	Temporal_Sup_L
33	21	−15	Insula_R
−45	−9	−12	Temporal_Sup_L
−45	−6	−12	Temporal_Sup_L
−15	57	27	Frontal_Sup_L
−12	60	27	Frontal_Sup_L
36	−72	51	Parietal_Sup_R

X, Y, and Z represent the spatial coordinates in the Montreal Neurological Institute space. R, right; L, left; Sup, superior; PD, Parkinson’s disease.

**Table 3:** ROI with significant differences in the PD and control groups.

Coordinates			Cluster Size	Labeling	Name
X	Y	Z			
−1	−41	−54	2	ROI1	Brainstem
17	−46	−45	2	ROI2	Right Cerebellum
30	−12	−45	1	ROI3	Right Temporal Fusiform Cortex
45	9	−21	1	ROI4	Right Temporal Pole: Superior Temporal Gyrus
−45	−7	−12	3	ROI5	Left Superior Temporal Gyrus
33	21	−15	1	ROI6	Right Insula
−14	59	27	2	ROI7	Left Frontal Pole
36	−72	51	1	ROI8	Right Superior Parietal Lobule
−33	−48	18	1	ROI9	Left Posterior Thalamic Radiation
−24	−47	26	4	ROI10	Left Posterior Corona Radiata
30	−42	24	1	ROI11	Right Posterior Corona Radiata
−24	−15	24	1	ROI12	Left Superior Corona Radiata
33	0	27	1	ROI13	Right Superior Longitudinal Fasciculus
30	−39	30	1	ROI14	Right Superior Longitudinal Fasciculus
−21	−24	36	1	ROI15	Left Superior Corona Radiata
21	−15	36	1	ROI16	Right Superior Corona Radiata

X, Y, and Z represent the spatial coordinates in the Montreal Neurological Institute space. ROI, region of interest; PD, Parkinson’s disease.

### 3.4. Analysis of white matter ROIs

**Figure 4:**
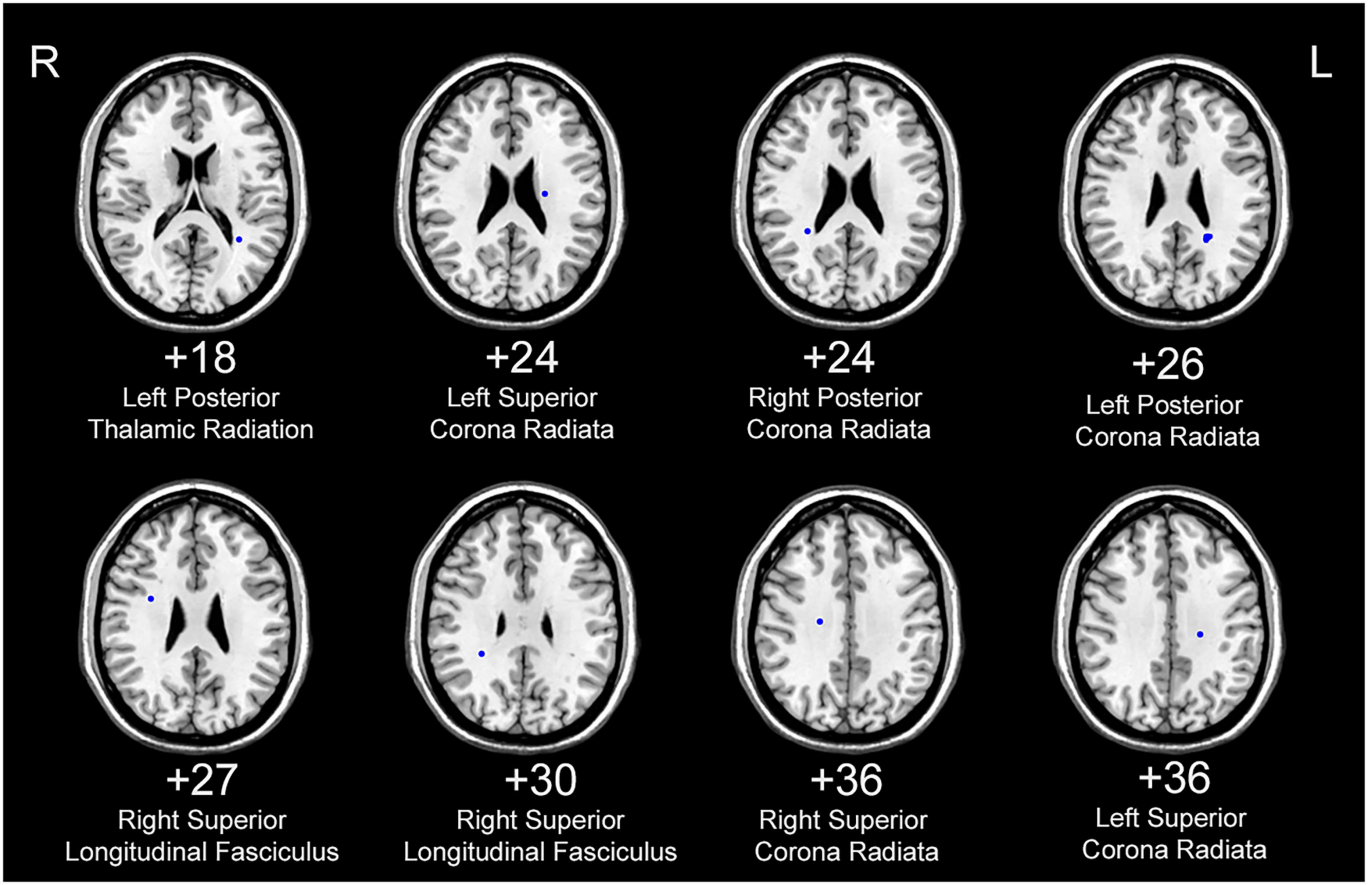
White matter areas with abnormal functional covariance connections. The areas marked in blue are the white matter areas, where the FCS is statistically different between the PD and HC groups (two sample *T*-test, *p* < 0.00005). The white figure depicts the cross-section level (Montreal Neurological Institute space). R: right side; L: left side. PD: Parkinsons’s disease, HC: healthy control.

The FCS of 11 white matter areas in the PD group differed from that of the control group (Table 4). They were mainly located in the left posterior thalamic radiation, bilateral posterior corona radiata, bilateral superior corona radiata, and right superior longitudinal fasciculus (Table 3; Figure 4).

**Table 4:** White matter brain areas with significant differences between the PD and control groups based on the John Hopkins University-White Matter atlas.

Coordinates			Brain region
X	Y	Z	
−33	−48	18	Left Posterior Thalamic Radiation
−24	−51	24	Left Posterior Corona Radiata
30	−42	24	Right Posterior Corona Radiata
−24	−15	24	Left Superior Corona Radiata
−21	−48	27	Left Posterior Corona Radiata
−21	−45	27	Left Posterior Corona Radiata
−24	−45	27	Left Posterior Corona Radiata
33	0	27	Right Superior Longitudinal Fasciculus
30	−39	30	Right Superior Longitudinal Fasciculus
−21	−24	36	Left Superior Corona Radiata
21	−15	36	Right Superior Corona Radiata

X, Y, and Z represent the spatial coordinates in the Montreal Neurological Institute space.

### 3.5. ALFF analysis based on cerebral gray matter

**Figure 5:**
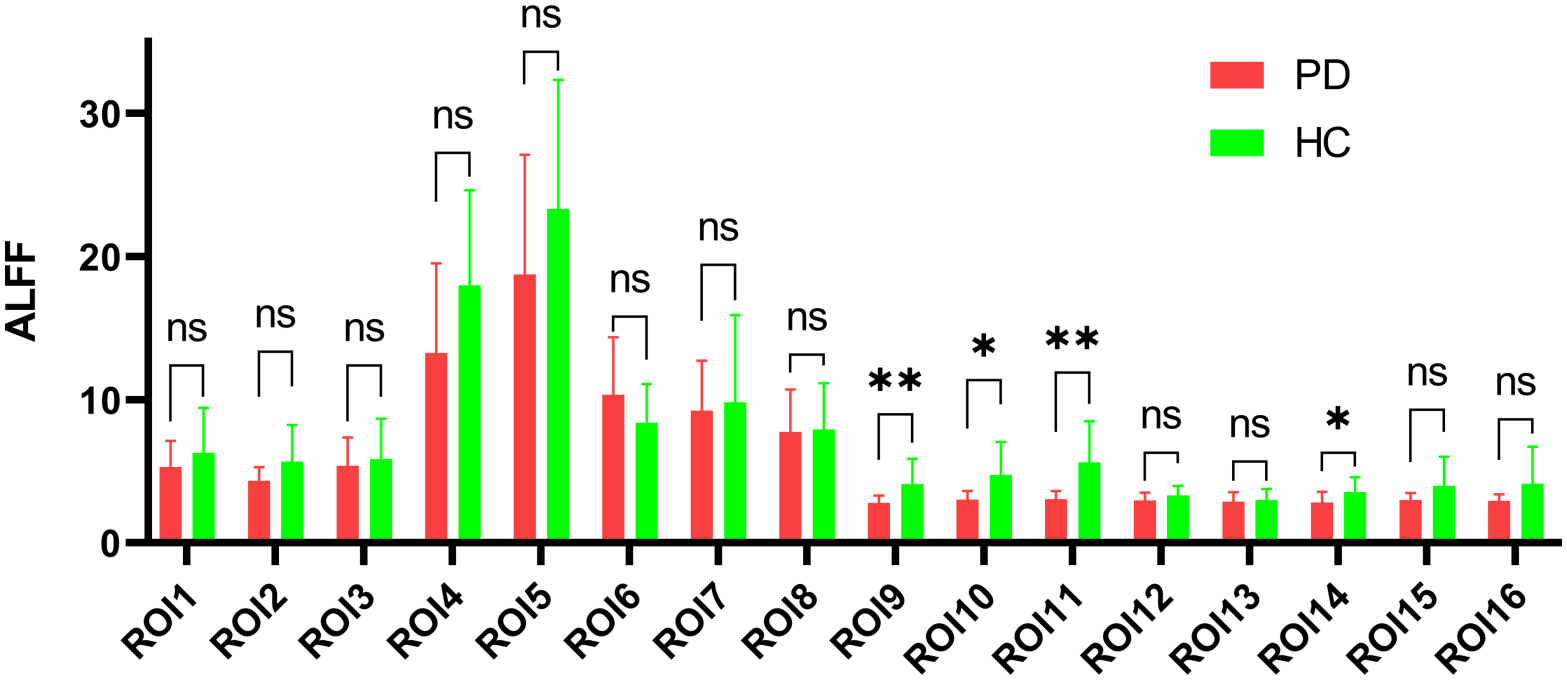
Amplitude comparison diagram of low-frequency fluctuation. ROI: region of interest, ALFF: amplitude of low-frequency fluctuations, PD: Parkinson’s disease group, HC: normal control group, ns: no significance, *p  <  0.05, **p  <  0.01.

**Figure 6:**
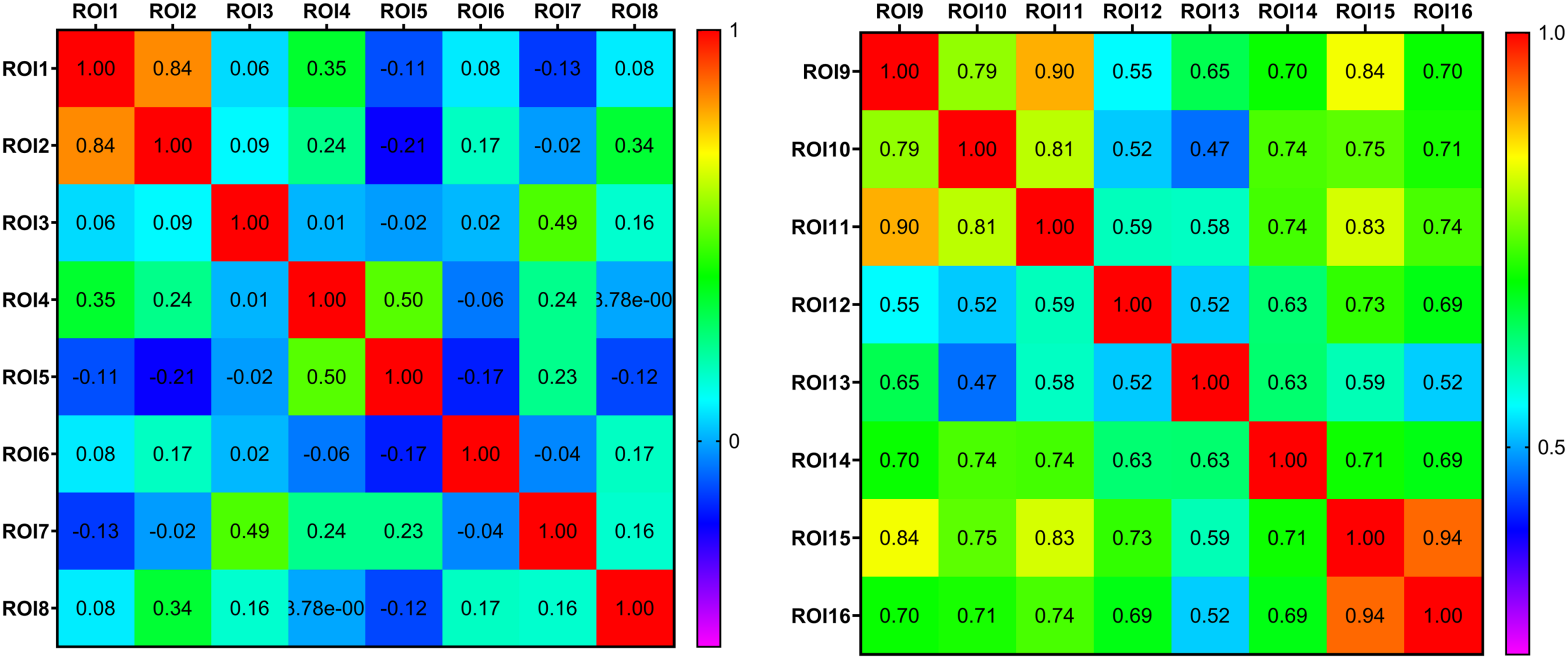
Thermodynamic diagram of the connectivity matrix of region of interest. Each row and column in the matrix corresponds to different region of interest. The value at the intersection of the rows and columns represents the correlation coefficient of the functional connection of the corresponding brain regions. Positive values represent a positive correlation and negative values represent a negative correlation. The larger the value, the closer the functional connection.

**Figure 7:**
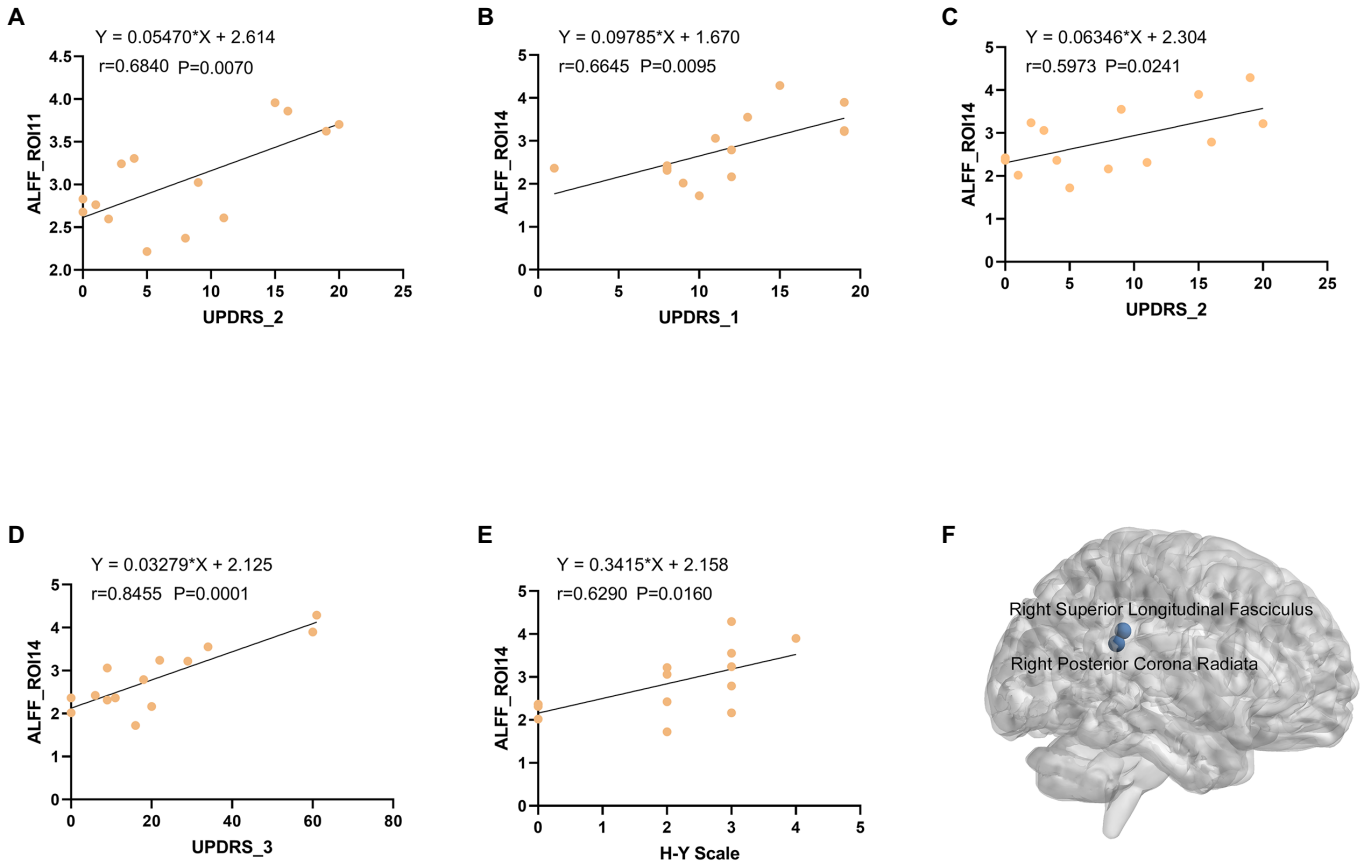
**(A)** The ALFF values of right ROI11 were positively correlated with UPDRS-2 (*r* = 0.5946, *p* = 0.0249). **(B)** The ALFF values of left ROI14 were positively correlated with UPDRS-1 (*r* = 0.5768, *p* = 0.0308). **(C)** The ALFF values of left ROI14 were positively correlated with UPDRS-2 (*r* = 0.5768, *p* = 0.0308). **(D)** The ALFF values of left ROI14 were positively correlated with UPDRS-3 (*r* = 0.5768, *p* = 0.0308). **(E)** The ALFF values of left ROI14 were positively correlated with H-Y Scale (*r* = 0.5768, *p* = 0.0308). **(F)** Three-dimensional map of the Right Posterior Corona Radiata (ROI11) and Right Superior Longitudinal Fasciculus (ROI14) brain regions. ALFF: amplitude of low-frequency fluctuations, ROI: region of interest, UPDRS: Unified Parkinson Disease Scale, H-Y Scale: Hoehn and Yahr Scale.

We analyzed the ALFF of 16 regions of interest. The ALFF of the normal group was generally higher than that of the PD group, and the ALFF of the insular brain region (ROI6) of the HC group was lower than that of the PD group. Significantly lower ALFF values were found in the left posterior thalamic radiation, bilateral posterior corona radiata and right superior longitudinal fasciculus than in the normal group (Figure 5). We analyzed the 8 gray matter region of interest correlation matrices and the 8 white matter region of interest correlation matrices separately, and found a strong correlation between the connection of the brainstem (ROI1) and right cerebellum (ROI2; Figure 6). We also analyzed the direct relationship between the region of interest and the Unified Parkinson’s Disease Rating Scale in the case group. We found that right posterior corona radiation (ROI11) showed a strong correlation with part 2 of the Unified Parkinson’s Disease Rating Scale, and right superior longitudinal fasciculus (ROI14) showed a strong correlation with parts 1, 2, and 3 of the Unified Parkinson’s Disease Rating Scale and Hoehn and Yahr Scale under the control of age and gender (Figure 7).

## 4. Discussion

Numerous neurodegenerative diseases are associated with the aging process, including the two most common ones, i.e., PD and AD. The establishment of biomarkers to improve early risk identification is essential for early treatment aimed at delaying or hindering the pathological process. Alterations in olfactory function are considered to be early biomarkers of neurodegenerative diseases (Dan et al., 2021). Olfactory dysfunction occurs in 90% of early PD and 85% of early AD cases, rendering it an attractive biomarker for the early diagnosis of these diseases. Olfactory decline is one of the principal early symptoms of PD. Mounting clinical and pathological data indicate that the dysfunction of the olfactory cortex may be responsible for the impairment of olfactory processing observed in PD. However, there is no clear evidence to establish a direct correlation between the changes in brain metabolism and hyposmia in PD (Baba et al., 2011).

The non-invasive nature and cost-effectiveness of olfactory function assessment make it extremely enticing option for the prediction and diagnosis of neurodegenerative diseases, because early diagnosis is essential for implementing interventions when the brain pathology is relatively closer to normal. Simple olfactory tests can effectively detect olfactory dysfunction in the two most common neurodegenerative diseases (Velayudhan et al., 2015; Morley et al., 2018), and help differentiate patients from normal individuals and patients with some similar characteristics but different etiopathologies (Duff et al., 2002). However, in some cases, olfactory testing alone may not be sufficient to determine certain specific diseases (Mesholam et al., 1998).

Olfactory testing should be used in conjunction with other disease-specific phenotypic tests to ensure accurate disease prediction and diagnosis. The combination of olfactory testing and dopamine transporter imaging predicted that 67% patients with prodromal PD underwent conversion to PD within 4 years (Jennings et al., 2017). Chen et al. showed that hyposmia and hyperechogenicity of the substantia nigra are important risk indicators for PD, and the combination of the two can improve the diagnostic specificity of patients with PD and those with primary tremor (Chen et al., 2012). The use of the L-DOPA challenge in combination with olfactory testing can improve the diagnostic sensitivity for early PD with mild movement disorder (Terroba Chambi et al., 2017).

Our previous research with a public database showed that (Wang et al., 2022) the dorsolateral prefrontal, anterior entorhinal cortex, and fronto-orbital cortices in the gray matter had abnormal connectivity with the posterior corona radiata and superior corona radiata in the white matter of patients with Parkinson’s hyposmia. The FCS of the right dorsolateral prefrontal cortex and white matter, and the covariance connection strength of the left superior corona radiata and gray matter function possess potential diagnostic value. To verify these findings, the current study analyzed the functional magnetic resonance data of 14 patients with Parkinson’s disease and 13 normal controls. The FCS of 16 region of interest (ROIs) in the PD group differed from that of the control group (Figure 7). The FCS of the posterior thalamic radiation, posterior corona radiata, anterior corona radiata, and white matter area of the superior longitudinal bundle were different. Correlation matrix analysis based on ALFF showed that the connection between the brainstem (ROI1) and right cerebellum (ROI2; Figure 6). In the partial correlation analysis, right posterior corona radiation (ROI11) showed a strong correlation with part 2 of the Unified Parkinson’s Disease Rating Scale, and right superior longitudinal fasciculus (ROI14) showed a strong correlation with parts 1, 2, and 3 of the Unified Parkinson’s Disease Rating Scale and Hoehn and Yahr Scale (Figure 7). The ROI7 of our current study matched fronto-orbital cortices found in previous studies in terms of spatial location. This further validates our previous finding that Parkinson’s hyposmia is altered in a specific brain region that is associated with the prefrontal orbital cortex (Wang et al., 2022). Right posterior corona radiation (ROI11) is consistent with the posterior corona radiata reported in our previous study. Our previous study considered Parkinson’s disease olfactory-related brain regions with ROI11. The present finding that ROI14 is closely associated with multiple Parkinson’s disease clinical scales and that this brain region predicts disease severity has clinical significance. In addition, these targeted locations and functional connectivity could potentially provide some cues for the PD treatment in view of occupational therapy (Foster et al., 2021) and occupational neuroscience (Yan et al., 2016; Shi et al., 2017; Shi et al., 2017; Wang et al., 2017, 2018; Wu et al., 2020).

Neuroimaging studies have provided several potential biomarkers for research on neurodegenerative diseases. Measurement of the ALFF of the BOLD signal can effectively reflect local spontaneous neuronal activity (Zuo and Xing, 2014; Cao et al., 2022) with high repeatability and reliability, facilitating its application as an indicator of functional differences in a single region (Zuo et al., 2019). Wang et al. (2020) used ALFF measurement of resting-state fMRI data to investigate the alterations in ALFF in patients with PD. They found that the ALFF was reduced in the left cerebellum, right anterior cuneiform lobe, and left posterior central/supramarginal gyrus (PostC) in patients with PD, and a greater decrease in the ALFF was observed in the left pallidum and anterior central gyrus/PostC, and the left caudate nucleus/putamen showed a positive correlation with the disease course, similar to some results of the current study. Zhang et al. (2022) found that chronic hypoxia can lead to extensive cognitive impairment, in addition to a significant reduction in the ROI density of the left olfactory cortex, right medial superior orbitofrontal gyrus, bilateral insular lobes, left globus pallidus and temporal lobe. These findings also support the idea that changes in orbitofrontal gyrus brain function may be a promising imaging marker in Parkinson’s disease, consistent with our idea.

A systematic review of neurodegenerative changes on MRI in patients with olfactory impairment, mild cognitive impairment (MCI) or dementia found that (Yi et al., 2022) 17 (71%) of 24 studies reported changes in hippocampal volume, and 14 reported the correlation between hippocampal volume and olfactory performance. Two of four prospective studies (50%) reported the potential value of baseline hippocampal volume as a marker of dementia transformation in MCI. Of the 24 studies that reported the findings of olfactory fMRI, 5 (21%) emphasized the role of olfactory fMRI in identifying individuals with early cognitive decline. Donoshita et al. (2021) used 7-T fMRI to assess olfactory function in the human brain, and found that olfactory stimulation mainly activated the piriform cortex and orbitofrontal cortex, except the amygdala. The subjective odor intensity was significantly related to the average fMRI signal of the piriform cortex. The value of the frontal orbital cortex as a neuroimaging marker in Parkinson’s disease was again discovered on a high-resolution magnetic resonance scanner.

Although some progress has been made in the study of brain structure and functional magnetic resonance in PD in recent years, few researchers have focused on the relationship of the changes in white–gray matter connectivity with olfactory function. Increasing evidence shows that the BOLD signal in white matter undergoes stimulus-related synchronous changes in response to olfactory and other related stimuli, and the signal changes in white matter pathways in the brain show clear specificity (Ding et al., 2018). We used the white–gray matter FCS method to devise a new approach to study the early changes in olfactory network connectivity in neurodegenerative diseases. This method can be used to detect changes in the brain regions associated with olfactory function in patients with neurodegenerative diseases, including PD. We found that specific brain regions (viz. the brainstem, right cerebellum, right fusiform gyrus, right temporal pole, lower part of the left superior temporal gyrus, right insular lobe, upper part of the left superior temporal gyrus, left frontal pole, and right superior parietal gyrus) have special connections with white matter fiber bundles (posterior thalamic radiation, posterior corona radiata, anterior corona radiata, and superior longitudinal bundle) in patients with PD. The frontal orbital cortex may be the brain area affected by specific brain function changes in Parkinson’s hyposmia. This indicates that the characteristics of olfactory-related brain networks can be used as potential neuroimaging biomarkers for the changes in early structural connectivity in PD.

## 5. Limitations of this study and future prospects

This is the first clinical study to use the FCS method to study the connectivity between the white and gray matter to elucidate the changes in olfactory network connectivity in PD. Since all MRI equipment used in this test were 1.5-T scanners, there were certain limitations in image clarity and other parameters. Moreover, the sample population was small, the clinical data were not detailed, and comprehensive neuropsychological evaluation was not performed, which hindered the exploration of specific PD dysfunction. In the near future, we endeavor to use 3-T (even 5 or 7-T) high-resolution MRI scanning combined with olfactory testing and comprehensive neuropsychological assessment to gather a large sample, and subsequently, in the later stage, we will use new task states and multimodal techniques to further study olfactory network function of patients with PD. Future studies should combine specific olfactory testing and other biomarkers to perform comprehensive evaluations of olfactory function.

## 6. Conclusion

Central anosmia is a potential marker of the early prodrome and progression of PD. Resting-state fMRI studies have shown that the impairment of olfactory function in patients with early PD is related to abnormal changes in olfactory-related central structures. Our results found abnormal connections between specific gray matter areas (frontal cortex) related to olfaction and white matter fiber bundles (posterior corona radiata, anterior corona radiata) in Chinese patients with PD, and ALFF values were found in specific white matter brain regions [Right posterior corona radiation (ROI11) and superior longitudinal fasciculus (ROI14)] to predict disease severity, indicating that the characteristics of olfaction-related brain networks can be used as potential neuroimaging biomarkers of connectivity structure changes in PD. In the future, combining dynamic observation of olfactory function with other disease-specific diagnostic tests may help improve the accuracy and specificity of olfactory dysfunction in the diagnosis of neurodegenerative diseases.

## Data availability statement

The original contributions presented in the study are included in the article/supplementary material, further inquiries can be directed to the corresponding authors.

## Ethics statement

The studies involving human participants were reviewed and approved by the Ethics Committee of Suzhou Science and Technology Town Hospital. The patients/participants provided their written informed consent to participate in this study.

## Author contributions

ZC and NW played a critical role in conceptualizing this study. YqW, HW, SD, and GL collected and analyzed all data. YqW and HY contributed to writing the first draft of the paper. XL, YjW, JZ, and YW prepared the manuscript. All authors provided critical feedback on the manuscript. All authors have read and approved the submitted manuscript.

## Funding

This work was supported by the National Natural Science Foundation of China (No. 82001160), Special Project for Diagnosis and Treatment of Key Clinical Diseases in Suzhou (No. LCZX202029), Suzhou City Medical Device and New Medicine Clinical Trial Institutional Capacity Improvement Project (No. SLT202001), Suzhou Science and Technology Development Plan (No. SS2019048), Scientific research project of Gusu School, Nanjing Medical University (No. GSKY20210240), Research Projects on Aging Health of Lianyungang City (No. L202201), and Project of Huaguoshan Mountain Talent Plan—Doctors for Innovation and Entrepreneurship.

## Conflict of interest

The authors declare that the research was conducted in the absence of any commercial or financial relationships that could be construed as a potential conflict of interest.

## Publisher’s note

All claims expressed in this article are solely those of the authors and do not necessarily represent those of their affiliated organizations, or those of the publisher, the editors and the reviewers. Any product that may be evaluated in this article, or claim that may be made by its manufacturer, is not guaranteed or endorsed by the publisher.

## Footnotes

^1^ https://www.nitrc.org/projects/mricron

^2^ https://ww2.mathworks.cn/
